# (4,7,13,16,21,24-Hexaoxa-1,10-diaza­bicyclo­[8.8.8]hexa­cosa­ne)sodium perchlorate

**DOI:** 10.1107/S1600536809039683

**Published:** 2009-10-17

**Authors:** Ilia A. Guzei, Joe W. Su, Lara C. Spencer, Ronald R. Burnette

**Affiliations:** a Department of Chemistry, University of Wisconsin-Madison, 1101 University Ave., Madison, WI 53706, USA; b DR SUSS CORP, 6007 McLeod Dr, Las Vegas, NV 89120, USA; c School of Pharmacy, University of Wisconsin, 777 Highland Ave., Madison, WI 53705, USA

## Abstract

The title compound, [Na(C_18_H_36_N_2_O_6_)]ClO_4_, was isolated and crystallized to understand more fully the ligand’s binding specificity to cations. The cation and anion reside at an inter­section of crystallographic twofold and threefold axes. The carbon atoms in the cation are disordered over two positions in a 3:2 ratio, and the anion is equally disordered over two positions. The geometries of the cation and anion are typical. The compound packs in alternating sheets of discrete cations and anions stacked along the *c* axis, separated by a distance equal to one-sixth the length of the *c* axis.

## Related literature

For general background to the macrocyclic polyether 4,7,13,16,21,24-hexa­oxa-1,10-diaza-bicyclo­[8.8.8]hexa­cosane, see: Izatt *et al.* (1985 ▶); Tait *et al.* (1997 ▶); Varga *et al.* (1994 ▶); Trend *et al.* (1993 ▶); Hamacher *et al.* (1986 ▶); Su & Burnette (2008 ▶). For related structures, see: Belaj *et al.* (1997 ▶); Tehan *et al.* (1974 ▶). For a description of the Cambridge Structural Database, see: Allen (2002 ▶) and for *Mogul*, see: Bruno *et al.* (2004 ▶).
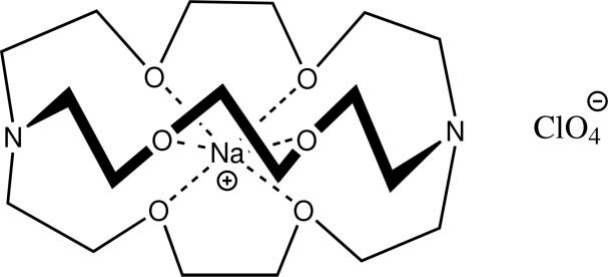


## Experimental

### 

#### Crystal data

[Na(C_18_H_36_N_2_O_6_)]ClO_4_
*M* *_r_* = 498.93
Rhombohedral, 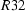

*a* = 8.4730 (3) Å
*c* = 28.220 (3) Å
*V* = 1754.5 (2) Å^3^
*Z* = 3
Mo- *K*α radiation
μ = 0.24 mm^−1^
*T* = 100 K
0.49 × 0.37 × 0.35 mm

#### Data collection

Bruker CCD-1000 area-detector diffractometer
Absorption correction: multi-scan (*SADABS*; Bruker, 2007 ▶) *T*_min_ = 0.893, *T*_max_ = 0.922
6885 measured reflections
805 independent reflections
765 reflections with *I* > 2σ(*I*)
*R*_int_ = 0.026

#### Refinement

*R*[*F* ^2^ > 2σ(*F* ^2^)] = 0.037
*wR*(*F* ^2^) = 0.113
*S* = 1.11
805 reflections
85 parameters
144 restraints
H-atom parameters constrained
Δρ_max_ = 0.28 e Å^−3^
Δρ_min_ = −0.18 e Å^−3^
Absolute structure: Flack (1983 ▶), 319 Friedel pairs
Flack parameter: 0.01 (15)

### 

Data collection: *SMART* (Bruker, 2007 ▶); cell refinement: *SAINT* (Bruker, 2007 ▶); data reduction: *SAINT*; program(s) used to solve structure: *SHELXTL* (Sheldrick, 2008 ▶); program(s) used to refine structure: *SHELXTL*; molecular graphics: *SHELXTL*, OLEX2 (Dolomanov *et al*., 2009 ▶) and *DIAMOND* (Brandenburg, 1999 ▶); software used to prepare material for publication: *SHELXTL*, *modiCIFer* (Guzei, 2007 ▶) and *publCIF* (Westrip, 2009 ▶).

## Supplementary Material

Crystal structure: contains datablocks global, I. DOI: 10.1107/S1600536809039683/hk2757sup1.cif

Structure factors: contains datablocks I. DOI: 10.1107/S1600536809039683/hk2757Isup2.hkl

Additional supplementary materials:  crystallographic information; 3D view; checkCIF report

## Figures and Tables

**Table 1:** Selected geometric parameters (Å, °)

Cl1—O2^i^	1.422 (4)
Cl1—O2	1.422 (4)
Cl1—O3^i^	1.434 (3)
Cl1—O3^ii^	1.434 (3)
Cl1—O3	1.434 (3)
Cl1—O3^iii^	1.434 (3)
Cl1—O3^iv^	1.434 (3)
Cl1—O3^v^	1.434 (3)
Na1—O1	2.5661 (15)
Na1—N1	2.684 (2)
O2—O3^iii^	1.639 (5)
O3—O3^v^	1.797 (10)

Symmetry codes: (i) 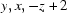; (ii) 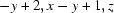; (iii) 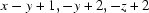; (iv) 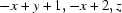; (v) 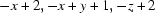.

**Fig. 1.**
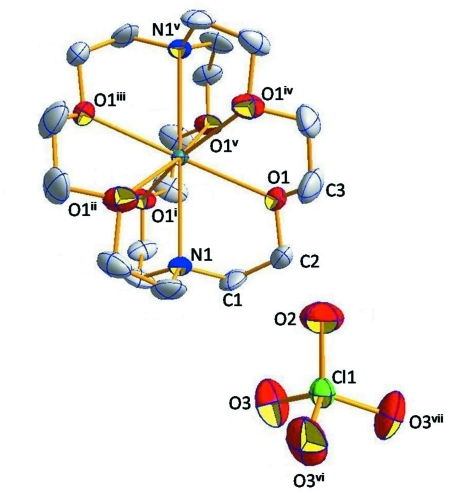
Molecular structure of (I). The thermal ellipsoids are shown at 50% probability level. Only the preferred orientation of the carbon atoms is shown and only tone orientation of the perchlorate molecule is shown. All hydrogen atoms were omitted for clarity. Symmetry transformations used to generate equivalent atoms: i: (-x + y+1,-x + 1,z) ii: (-y + 1,x-y,z) iii: (-x + 4/3,-x + y+2/3,-z + 5/3) iv: (y + 1/3,x - 1/3,-z + 5/3) v: (x-y + 1/3,-y + 2/3,-z + 5/3) vi: (-x + y+1,-x + 2,z) vii: (-y + 2,x-y + 1,z).

## supplementary crystallographic information

### Comment

The macrocyclic polyether 4,7,13,16,21,24-hexaoxa-1,10-diaza-bicyclo[8.8.8]hexacosane (222) is a classic example of a host molecule possessing important clinical functions. 222 encapsulates 1:1 various alkali and alkaline earth metals, and features high selectivity for K^+^ and Sr^2+^ in solution (Izatt *et al.*, 1985). Tait *et al.* (1997) formulated a cation exchange resin treated with 222 that sorbed more than 95% of the fallout nuclide ^90^Sr in liquid milk (295 K, pH 5.2, 4 h contact time, 1:50 resin to milk volume ratio). Varga *et al.* (1994) synthesized functionalized 222 for ^85^Sr^2+^ decorporation in the rat and mouse. J. E. Trend and co-workers (1993) formulated 222 with a covalently bound chromophore to assay clinical blood K^+4^. Hamacher *et al.* (1986) developed the use of [K^+^(222)]^18^F^-^ as a phase transfer catalyst in synthesizing the clinically significant PET tracer 2-[^18^*F*]fluoro-2-deoxy-D-glucose. Due to 222 obvious industrial and clinical applications the relative binding characteristics of 222 to Li^+^, Na^+^ and K^+^ have been studied in order to more fully understand 222's binding specificity to cations (Su & Burnette, 2008).

In the title compound, (I), both the Na^+^(222) cation and the perchlorate anion of (I) reside at an intersection of crystallographic twofold and threefold axes. All the carbon atoms in the cation are disordered over two positions in a 3:2 ratio. The perchlorate anion is equally disordered over two positions. Multiple restraints were applied to ensure computational stability of the refinement.

The bond distances and angles within (I) are typical as confirmed by the *Mogul* structural check (Bruno *et. al*, 2004). Among 59 relevant compounds reported to the CSD (Allen, 2002), the most closely related is (4,7,13,16,21,24-hexaoxa-1,10-diazabicyclo(8.8.8)hexacosane)-sodium periodate which contains the same cation as (I) and a periodate anion instead of the perchlorate anion (Belaj *et al.*, 1997), and sodium (2,2,2)-crypt-sodium (Tehan *et al.*, 1974) which forms crystals in the same rhombohedral space group R32, as (I).

The packing structure of compound (I) consists of alternating sheets of cations and anions stacked along the *c* axis. The distance between these sheets is 4.70 Å, or one sixth of the length of the *c* axis.

### Experimental

An equimolar mixture of 222 and NaClO_4_ was prepared in acetone. The mixture was allowed to evaporate slowly at room temperature until crystallization was observed.

### Refinement

All H-atoms were placed in idealized locations and refined as riding with appropriate thermal displacement coefficients *U*_iso_(H) = 1.2 times *U*_eq_(bearing atom).

The following restraints (expressed as *SHELXL* commands) were used. Thus, we imposed distance similarity restraints on the C—C and C—N bonds involving disordered atoms and refined the ClO_4_^-^ anion with an idealized geometry allowing the Cl—O distanct to refine as a free variable. The thermal displacement parameters for C3 and C3a were restrained to approximate isotropic behavior.

EQIV $3 Y+1/3, X-1/3, –*Z*+5/3 SADI 0.005 C1 C2 C1A C2A C3 C3_$3 C3A C3A_$3 SADI 0.005 N1 C1 N1 C1A SADI 0.005 O1 C2 O1 C2A O1 C3 O1 C3A *DFIX* 21 0.005 C L1 O3 CL1 O2 *DFIX* 21.633 0.005 O2 O3 SIMU DELU ISOR 0.02 C3 C3A FVAR 0.49309 1.43008 0.34032

### Crystal data

**Table:**

[Na(C_18_H_36_N_2_O_6_)]ClO_4_	*F*(000) = 798
*M**_r_* = 498.93	*D*_x_ = 1.417 Mg m^−^^3^
Rhombohedral, *R*32	Mo *K*α radiation, λ = 0.71073 Å
Hall symbol: R 3 2"	Cell parameters from 999 reflections
*a* = 8.4730 (3) Å	θ = 2.2–26.4°
*c* = 28.220 (3) Å	µ = 0.24 mm^−^^1^
α = 90°	*T* = 100 K
γ = 120°	Block, colorless
*V* = 1754.5 (2) Å^3^	0.49 × 0.37 × 0.35 mm
*Z* = 3	

### Data collection

**Table:**

Bruker CCD-1000 area-detector diffractometer	805 independent reflections
Radiation source: fine-focus sealed tube	765 reflections with *I* > 2σ(*I*)
graphite	*R*_int_ = 0.026
0.30° ω scans	θ_max_ = 26.4°, θ_min_ = 2.2°
Absorption correction: multi-scan (*SADABS*; Bruker, 2007)	*h* = −10→10
*T*_min_ = 0.893, *T*_max_ = 0.922	*k* = −10→10
6885 measured reflections	*l* = −35→35

### Refinement

**Table:**

Refinement on *F*^2^	Secondary atom site location: difference Fourier map
Least-squares matrix: full	Hydrogen site location: inferred from neighbouring sites
*R*[*F*^2^ > 2σ(*F*^2^)] = 0.037	H-atom parameters constrained
*wR*(*F*^2^) = 0.113	*w* = 1/[σ^2^(*F*_o_^2^) + (0.0715*P*)^2^ + 1.1083*P*] where *P* = (*F*_o_^2^ + 2*F*_c_^2^)/3
*S* = 1.11	(Δ/σ)_max_ < 0.001
805 reflections	Δρ_max_ = 0.28 e Å^−^^3^
85 parameters	Δρ_min_ = −0.18 e Å^−^^3^
144 restraints	Absolute structure: Flack (1983), 319 Friedel pairs
Primary atom site location: structure-invariant direct methods	Flack parameter: 0.01 (15)

### Special details

**Table:**

Geometry. All e.s.d.'s (except the e.s.d. in the dihedral angle between two l.s. planes) are estimated using the full covariance matrix. The cell e.s.d.'s are taken into account individually in the estimation of e.s.d.'s in distances, angles and torsion angles; correlations between e.s.d.'s in cell parameters are only used when they are defined by crystal symmetry. An approximate (isotropic) treatment of cell e.s.d.'s is used for estimating e.s.d.'s involving l.s. planes.
Refinement. Refinement of *F*^2^ against ALL reflections. The weighted *R*-factor *wR* and goodness of fit *S* are based on *F*^2^, conventional *R*-factors *R* are based on *F*, with *F* set to zero for negative *F*^2^. The threshold expression of *F*^2^ > σ(*F*^2^) is used only for calculating *R*-factors(gt) *etc*. and is not relevant to the choice of reflections for refinement. *R*-factors based on *F*^2^ are statistically about twice as large as those based on *F*, and *R*- factors based on ALL data will be even larger.

### Fractional atomic coordinates and isotropic or equivalent isotropic displacement parameters (Å^2^)

**Table:**

	*x*	*y*	*z*	*U*_iso_*/*U*_eq_	Occ. (<1)
Cl1	1.0000	1.0000	1.0000	0.0347 (3)	
Na1	0.6667	0.3333	0.8333	0.0246 (3)	
O1	0.9844 (2)	0.5262 (3)	0.86993 (4)	0.0589 (6)	
O2	1.0000	1.0000	0.94963 (15)	0.0768 (17)	0.50
O3	0.8972 (8)	0.8167 (4)	1.01732 (13)	0.0762 (10)	0.50
N1	0.6667	0.3333	0.92846 (8)	0.0420 (6)	
C1	0.8527 (6)	0.3802 (11)	0.9424 (2)	0.0688 (15)	0.60
H1A	0.8634	0.3967	0.9772	0.083*	0.60
H1B	0.8694	0.2754	0.9348	0.083*	0.60
C1A	0.8443 (7)	0.4767 (11)	0.9471 (3)	0.0534 (16)	0.40
H1C	0.8525	0.5973	0.9452	0.064*	0.40
H1D	0.8581	0.4518	0.9807	0.064*	0.40
C2	1.0022 (10)	0.5443 (10)	0.92024 (13)	0.0614 (18)	0.60
H2A	0.9972	0.6535	0.9305	0.074*	0.60
H2B	1.1210	0.5592	0.9301	0.074*	0.60
C2A	0.9880 (17)	0.475 (3)	0.9180 (2)	0.087 (4)	0.40
H2C	1.1084	0.5602	0.9319	0.104*	0.40
H2D	0.9725	0.3514	0.9186	0.104*	0.40
C3	1.0622 (10)	0.7167 (5)	0.85941 (11)	0.087 (2)	0.60
H3A	1.1909	0.7854	0.8699	0.105*	0.60
H3B	0.9934	0.7671	0.8756	0.105*	0.60
C3A	1.1033 (9)	0.6727 (12)	0.8398 (4)	0.094 (4)	0.40
H3C	1.1327	0.6254	0.8110	0.113*	0.40
H3D	1.2183	0.7554	0.8565	0.113*	0.40

### Atomic displacement parameters (Å^2^)

**Table:**

	*U*^11^	*U*^22^	*U*^33^	*U*^12^	*U*^13^	*U*^23^
Cl1	0.0316 (3)	0.0316 (3)	0.0408 (5)	0.01580 (17)	0.000	0.000
Na1	0.0265 (4)	0.0265 (4)	0.0208 (6)	0.0132 (2)	0.000	0.000
O1	0.0367 (7)	0.0832 (14)	0.0448 (8)	0.0211 (8)	−0.0063 (6)	0.0004 (7)
O2	0.093 (3)	0.093 (3)	0.045 (3)	0.0464 (14)	0.000	0.000
O3	0.071 (3)	0.0472 (18)	0.109 (3)	0.028 (2)	0.015 (3)	0.0307 (17)
N1	0.0486 (9)	0.0486 (9)	0.0289 (10)	0.0243 (5)	0.000	0.000
C1	0.068 (3)	0.087 (4)	0.037 (2)	0.028 (3)	−0.026 (2)	0.003 (3)
C1A	0.060 (4)	0.058 (4)	0.032 (3)	0.022 (4)	−0.017 (3)	0.000 (3)
C2	0.040 (3)	0.077 (4)	0.047 (3)	0.014 (2)	−0.008 (2)	−0.0187 (19)
C2A	0.046 (4)	0.146 (12)	0.054 (5)	0.037 (6)	−0.025 (3)	0.008 (5)
C3	0.067 (4)	0.056 (3)	0.088 (4)	−0.007 (3)	−0.033 (3)	0.000 (3)
C3A	0.041 (4)	0.100 (7)	0.089 (6)	−0.005 (4)	0.009 (4)	−0.004 (5)

### Geometric parameters (Å, °)

**Table:**

Cl1—O2^i^	1.422 (4)	O3—O3^i^	1.533 (9)
Cl1—O2	1.422 (4)	O3—O3^v^	1.797 (10)
Cl1—O3^i^	1.434 (3)	N1—C1	1.474 (4)
Cl1—O3^ii^	1.434 (3)	N1—C1^vi^	1.474 (4)
Cl1—O3	1.434 (3)	N1—C1^viii^	1.474 (4)
Cl1—O3^iii^	1.434 (3)	N1—C1A^vi^	1.480 (4)
Cl1—O3^iv^	1.434 (3)	N1—C1A	1.480 (4)
Cl1—O3^v^	1.434 (3)	N1—C1A^viii^	1.480 (4)
Na1—O1	2.5661 (15)	C1—C2	1.473 (5)
Na1—O1^vi^	2.5661 (15)	C1—H1A	0.9900
Na1—O1^vii^	2.5661 (15)	C1—H1B	0.9900
Na1—O1^viii^	2.5661 (15)	C1A—C2A	1.476 (6)
Na1—O1^ix^	2.5661 (15)	C1A—H1C	0.9900
Na1—O1^x^	2.5661 (15)	C1A—H1D	0.9900
Na1—N1	2.684 (2)	C2—H2A	0.9900
Na1—N1^ix^	2.685 (2)	C2—H2B	0.9900
O1—C2	1.428 (4)	C2A—H2C	0.9900
O1—C2A	1.428 (4)	C2A—H2D	0.9900
O1—C3	1.436 (4)	C3—C3^ix^	1.483 (6)
O1—C3A	1.425 (5)	C3—H3A	0.9900
O2—O3^iii^	1.639 (5)	C3—H3B	0.9900
O2—O3^v^	1.639 (5)	C3A—C3A^ix^	1.474 (6)
O2—O3^i^	1.639 (5)	C3A—H3C	0.9900
O3—O2^i^	1.639 (5)	C3A—H3D	0.9900
			
O2^i^—Cl1—O2	180.000 (4)	C3—O1—Na1	112.2 (3)
O2^i^—Cl1—O3^i^	109.92 (16)	Cl1—O2—O3^iii^	55.32 (15)
O2—Cl1—O3^i^	70.08 (16)	Cl1—O2—O3^v^	55.32 (15)
O2^i^—Cl1—O3^ii^	70.08 (16)	O3^iii^—O2—O3^v^	90.8 (2)
O2—Cl1—O3^ii^	109.92 (16)	Cl1—O2—O3^i^	55.32 (15)
O3^i^—Cl1—O3^ii^	172.5 (5)	O3^iii^—O2—O3^i^	90.8 (2)
O2^i^—Cl1—O3	70.08 (16)	O3^v^—O2—O3^i^	90.8 (2)
O2—Cl1—O3	109.92 (16)	Cl1—O3—O3^i^	57.7 (2)
O3^i^—Cl1—O3	64.6 (5)	Cl1—O3—O2^i^	54.60 (16)
O3^ii^—Cl1—O3	109.01 (16)	O3^i^—O3—O2^i^	94.9 (4)
O2^i^—Cl1—O3^iii^	109.92 (16)	Cl1—O3—O3^v^	51.2 (2)
O2—Cl1—O3^iii^	70.08 (16)	O3^i^—O3—O3^v^	88.7 (3)
O3^i^—Cl1—O3^iii^	109.01 (16)	O2^i^—O3—O3^v^	85.6 (3)
O3^ii^—Cl1—O3^iii^	77.6 (5)	C1—N1—C1^vi^	113.1 (2)
O3—Cl1—O3^iii^	172.5 (5)	C1—N1—C1^viii^	113.1 (2)
O2^i^—Cl1—O3^iv^	70.08 (16)	C1^vi^—N1—C1^viii^	113.1 (2)
O2—Cl1—O3^iv^	109.92 (16)	C1A^vi^—N1—C1A	108.0 (3)
O3^i^—Cl1—O3^iv^	77.6 (5)	C1A^vi^—N1—C1A^viii^	108.0 (3)
O3^ii^—Cl1—O3^iv^	109.01 (16)	C1A—N1—C1A^viii^	108.0 (3)
O3—Cl1—O3^iv^	109.01 (16)	C1—N1—Na1	105.5 (2)
O3^iii^—Cl1—O3^iv^	64.6 (4)	C1^vi^—N1—Na1	105.5 (3)
O2^i^—Cl1—O3^v^	109.92 (16)	C1^viii^—N1—Na1	105.5 (2)
O2—Cl1—O3^v^	70.08 (16)	C1A^vi^—N1—Na1	110.9 (3)
O3^i^—Cl1—O3^v^	109.01 (16)	C1A—N1—Na1	110.9 (3)
O3^ii^—Cl1—O3^v^	64.6 (5)	C1A^viii^—N1—Na1	110.9 (3)
O3—Cl1—O3^v^	77.6 (5)	C2—C1—N1	116.1 (6)
O3^iii^—Cl1—O3^v^	109.01 (16)	C2—C1—H1A	108.3
O3^iv^—Cl1—O3^v^	172.5 (5)	N1—C1—H1A	108.3
O1—Na1—O1^vi^	104.90 (3)	C2—C1—H1B	108.3
O1—Na1—O1^vii^	167.11 (9)	N1—C1—H1B	108.3
O1^vi^—Na1—O1^vii^	86.11 (9)	H1A—C1—H1B	107.4
O1—Na1—O1^viii^	104.90 (3)	C2A—C1A—N1	107.4 (8)
O1^vi^—Na1—O1^viii^	104.90 (3)	C2A—C1A—H1C	110.2
O1^vii^—Na1—O1^viii^	65.09 (7)	N1—C1A—H1C	110.2
O1—Na1—O1^ix^	65.09 (7)	C2A—C1A—H1D	110.2
O1^vi^—Na1—O1^ix^	167.11 (9)	N1—C1A—H1D	110.2
O1^vii^—Na1—O1^ix^	104.90 (3)	H1C—C1A—H1D	108.5
O1^viii^—Na1—O1^ix^	86.11 (9)	O1—C2—C1	109.0 (4)
O1—Na1—O1^x^	86.11 (9)	O1—C2—H2A	109.9
O1^vi^—Na1—O1^x^	65.09 (7)	C1—C2—H2A	109.9
O1^vii^—Na1—O1^x^	104.89 (3)	O1—C2—H2B	109.9
O1^viii^—Na1—O1^x^	167.11 (9)	C1—C2—H2B	109.9
O1^ix^—Na1—O1^x^	104.89 (3)	H2A—C2—H2B	108.3
O1—Na1—N1	66.27 (3)	O1—C2A—C1A	112.6 (7)
O1^vi^—Na1—N1	66.27 (3)	O1—C2A—H2C	109.1
O1^vii^—Na1—N1	113.73 (3)	C1A—C2A—H2C	109.1
O1^viii^—Na1—N1	66.27 (3)	O1—C2A—H2D	109.1
O1^ix^—Na1—N1	113.73 (3)	C1A—C2A—H2D	109.1
O1^x^—Na1—N1	113.73 (3)	H2C—C2A—H2D	107.8
O1—Na1—N1^ix^	113.73 (3)	O1—C3—C3^ix^	105.9 (4)
O1^vi^—Na1—N1^ix^	113.73 (3)	O1—C3—H3A	110.5
O1^vii^—Na1—N1^ix^	66.27 (3)	C3^ix^—C3—H3A	110.5
O1^viii^—Na1—N1^ix^	113.73 (3)	O1—C3—H3B	110.5
O1^ix^—Na1—N1^ix^	66.27 (3)	C3^ix^—C3—H3B	110.5
O1^x^—Na1—N1^ix^	66.27 (3)	H3A—C3—H3B	108.7
N1—Na1—N1^ix^	180.0	O1—C3A—C3A^ix^	106.5 (5)
C3A—O1—C2A	135.9 (9)	O1—C3A—H3C	110.4
C2—O1—C3	96.9 (4)	C3A^ix^—C3A—H3C	110.4
C3A—O1—Na1	112.0 (4)	O1—C3A—H3D	110.4
C2A—O1—Na1	111.3 (6)	C3A^ix^—C3A—H3D	110.4
C2—O1—Na1	119.3 (3)	H3C—C3A—H3D	108.6
			
O3^i^—Cl1—O2—O3^iii^	120.000 (5)	O1^x^—Na1—O1—C3	126.9 (3)
O3^ii^—Cl1—O2—O3^iii^	−68.0 (5)	N1—Na1—O1—C3	−115.1 (3)
O3—Cl1—O2—O3^iii^	172.0 (5)	N1^ix^—Na1—O1—C3	64.9 (3)
O3^iv^—Cl1—O2—O3^iii^	52.0 (5)	O1—Na1—N1—C1	−23.0 (4)
O3^v^—Cl1—O2—O3^iii^	−120.000 (5)	O1^vi^—Na1—N1—C1	97.0 (4)
O3^i^—Cl1—O2—O3^v^	−120.000 (14)	O1^vii^—Na1—N1—C1	171.1 (3)
O3^ii^—Cl1—O2—O3^v^	52.0 (5)	O1^viii^—Na1—N1—C1	−143.0 (4)
O3—Cl1—O2—O3^v^	−68.0 (5)	O1^ix^—Na1—N1—C1	−68.9 (3)
O3^iii^—Cl1—O2—O3^v^	120.000 (13)	O1^x^—Na1—N1—C1	51.1 (3)
O3^iv^—Cl1—O2—O3^v^	172.0 (5)	O1—Na1—N1—C1^vi^	−143.0 (3)
O3^ii^—Cl1—O2—O3^i^	172.0 (5)	O1^vi^—Na1—N1—C1^vi^	−23.0 (3)
O3—Cl1—O2—O3^i^	52.0 (5)	O1^vii^—Na1—N1—C1^vi^	51.1 (3)
O3^iii^—Cl1—O2—O3^i^	−120.000 (3)	O1^viii^—Na1—N1—C1^vi^	97.0 (3)
O3^iv^—Cl1—O2—O3^i^	−68.0 (5)	O1^ix^—Na1—N1—C1^vi^	171.1 (3)
O3^v^—Cl1—O2—O3^i^	120.000 (2)	O1^x^—Na1—N1—C1^vi^	−68.9 (3)
O2^i^—Cl1—O3—O3^i^	125.0 (4)	O1—Na1—N1—C1^viii^	97.0 (4)
O2—Cl1—O3—O3^i^	−55.0 (4)	O1^vi^—Na1—N1—C1^viii^	−143.0 (4)
O3^ii^—Cl1—O3—O3^i^	−175.6 (3)	O1^vii^—Na1—N1—C1^viii^	−68.9 (4)
O3^iv^—Cl1—O3—O3^i^	65.5 (5)	O1^viii^—Na1—N1—C1^viii^	−23.0 (4)
O3^v^—Cl1—O3—O3^i^	−118.2 (4)	O1^ix^—Na1—N1—C1^viii^	51.1 (4)
O2—Cl1—O3—O2^i^	180.000 (4)	O1^x^—Na1—N1—C1^viii^	171.1 (4)
O3^i^—Cl1—O3—O2^i^	−125.0 (4)	O1—Na1—N1—C1A^vi^	−107.5 (4)
O3^ii^—Cl1—O3—O2^i^	59.45 (19)	O1^vi^—Na1—N1—C1A^vi^	12.5 (4)
O3^iv^—Cl1—O3—O2^i^	−59.45 (19)	O1^vii^—Na1—N1—C1A^vi^	86.6 (4)
O3^v^—Cl1—O3—O2^i^	116.8 (3)	O1^viii^—Na1—N1—C1A^vi^	132.5 (4)
O2^i^—Cl1—O3—O3^v^	−116.8 (3)	O1^ix^—Na1—N1—C1A^vi^	−153.4 (4)
O2—Cl1—O3—O3^v^	63.2 (3)	O1^x^—Na1—N1—C1A^vi^	−33.4 (4)
O3^i^—Cl1—O3—O3^v^	118.2 (4)	O1—Na1—N1—C1A	12.5 (4)
O3^ii^—Cl1—O3—O3^v^	−57.3 (4)	O1^vi^—Na1—N1—C1A	132.5 (4)
O3^iv^—Cl1—O3—O3^v^	−176.2 (2)	O1^vii^—Na1—N1—C1A	−153.4 (4)
O1^vi^—Na1—O1—C3A	153.1 (4)	O1^viii^—Na1—N1—C1A	−107.5 (4)
O1^vii^—Na1—O1—C3A	−58.9 (4)	O1^ix^—Na1—N1—C1A	−33.4 (4)
O1^viii^—Na1—O1—C3A	−96.7 (4)	O1^x^—Na1—N1—C1A	86.6 (4)
O1^ix^—Na1—O1—C3A	−18.3 (4)	O1—Na1—N1—C1A^viii^	132.5 (5)
O1^x^—Na1—O1—C3A	90.1 (4)	O1^vi^—Na1—N1—C1A^viii^	−107.5 (5)
N1—Na1—O1—C3A	−151.8 (4)	O1^vii^—Na1—N1—C1A^viii^	−33.4 (4)
N1^ix^—Na1—O1—C3A	28.2 (4)	O1^viii^—Na1—N1—C1A^viii^	12.5 (5)
O1^vi^—Na1—O1—C2A	−35.5 (7)	O1^ix^—Na1—N1—C1A^viii^	86.6 (5)
O1^vii^—Na1—O1—C2A	112.5 (7)	O1^x^—Na1—N1—C1A^viii^	−153.4 (5)
O1^viii^—Na1—O1—C2A	74.8 (7)	C3—O1—C2—C1	149.0 (7)
O1^ix^—Na1—O1—C2A	153.2 (7)	Na1—O1—C2—C1	28.7 (9)
O1^x^—Na1—O1—C2A	−98.4 (7)	C3A—O1—C2A—C1A	115.5 (10)
N1—Na1—O1—C2A	19.7 (7)	Na1—O1—C2A—C1A	−53.0 (13)
N1^ix^—Na1—O1—C2A	−160.3 (7)	C2—O1—C3—C3^ix^	−177.7 (7)
O1^vi^—Na1—O1—C2	−58.0 (5)	Na1—O1—C3—C3^ix^	−52.1 (8)
O1^vii^—Na1—O1—C2	90.0 (5)	C2A—O1—C3A—C3A^ix^	−116.9 (11)
O1^viii^—Na1—O1—C2	52.3 (5)	Na1—O1—C3A—C3A^ix^	51.6 (10)
O1^ix^—Na1—O1—C2	130.7 (5)	C1^vi^—N1—C1—C2	166.0 (5)
O1^x^—Na1—O1—C2	−120.9 (5)	C1^viii^—N1—C1—C2	−63.7 (9)
N1—Na1—O1—C2	−2.8 (5)	Na1—N1—C1—C2	51.2 (6)
N1^ix^—Na1—O1—C2	177.2 (5)	C1A^vi^—N1—C1A—C2A	79.9 (10)
O1^vi^—Na1—O1—C3	−170.2 (3)	C1A^viii^—N1—C1A—C2A	−163.5 (7)
O1^vii^—Na1—O1—C3	−22.2 (3)	Na1—N1—C1A—C2A	−41.8 (8)
O1^viii^—Na1—O1—C3	−59.9 (3)	N1—C1—C2—O1	−55.6 (9)
O1^ix^—Na1—O1—C3	18.5 (3)	N1—C1A—C2A—O1	65.1 (14)

Symmetry codes: (i) *y*, *x*, −*z*+2; (ii) −*y*+2, *x*−*y*+1, *z*; (iii) *x*−*y*+1, −*y*+2, −*z*+2; (iv) −*x*+*y*+1, −*x*+2, *z*; (v) −*x*+2, −*x*+*y*+1, −*z*+2; (vi) −*x*+*y*+1, −*x*+1, *z*; (vii) −*x*+4/3, −*x*+*y*+2/3, −*z*+5/3; (viii) −*y*+1, *x*−*y*, *z*; (ix) *y*+1/3, *x*−1/3, −*z*+5/3; (x) *x*−*y*+1/3, −*y*+2/3, −*z*+5/3.
